# Protocol for optimized methylation context-sensitive enzyme ddRAD library preparation from partially degraded genomic plant DNA

**DOI:** 10.1016/j.xpro.2026.104778

**Published:** 2026-08-11

**Authors:** César Herrero-González, Sylvia Sagen Johnsen, Rómulo Sacramento Sobral, Marian Schubert

**Affiliations:** 1 Department of Plant Sciences, Norwegian University of Life Sciences (NMBU), Ås 1433, Norway; 2 Natural History Museum, University of Oslo, Research Group Evolution, eDNA, Genomics and Ethnobotany, 0562 Oslo, Norway

**Keywords:** Genetics, Sequencing, Plant sciences

## Abstract

Here, we present an optimized methylation context-sensitive enzyme ddRAD (MCSeEd) library-preparation protocol for methylation analysis in CG, CHG, and CHH sequence contexts from plant samples that yield low-quality or partially degraded genomic DNA. We describe steps for quadruple restriction-enzyme digestion and adapter ligation, double size selection, freeze-and-squeeze gel purification, and bead-based cleanup. We then detail a two-step PCR enrichment procedure to generate Illumina-compatible epigenomic libraries.

## Highlights

• Simplified restriction-ligation using a common enzyme buffer
• Instructions for dual size selection and gel-based DNA recovery
• Two-step PCR enrichment and TapeStation library quality assessment

## Abstract (editor highlights)

Publisher’s note: Undertaking any experimental protocol requires adherence to local institutional guidelines for laboratory safety and ethics.

## Graphical abstract

**Figure:**
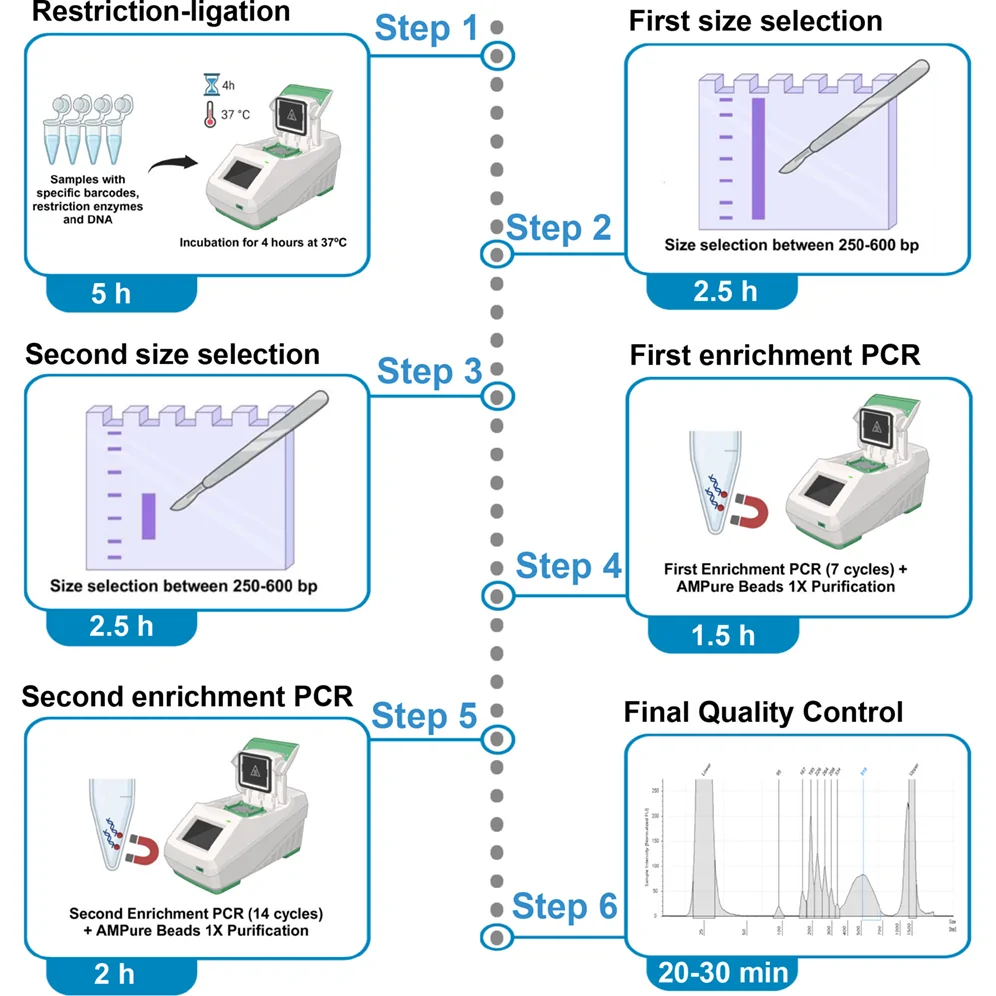

## Before you begin

This protocol describes an optimized MCSeEd library preparation workflow for low-quality or partially degraded genomic DNA samples from plants. Common features of such samples include DNA fragmentation, visible as smearing on agarose gels, and the presence of co-extracted contaminants common in plant tissues, such as phenols and carbohydrates which can interfere with downstream enzymatic reactions.

The protocol is based on the approach developed by Di Marsico and collaborators^1^ (Figure S1) and is adapted to improve fragment recovery and final library quality when high-integrity plant DNA is difficult to obtain.^2^ Genomic DNA, barcoded adapters and all required reagents should be prepared as described below before starting the protocol.

### Innovation

This protocol extends from the original MCSeEd workflow by enabling epigenomic library preparation from plant genomic DNA of suboptimal quality or integrity. This limitation is particularly relevant for the species used here, *Nardus stricta*, whose narrow, tightly enrolled, rigid, coriaceous and silica-rich leaf blades may compromise efficient tissue homogenization.^3^^,^^4^ Similar constraints can also arise from plant samples containing co-extracted contaminants, such as phenolic compounds, polysaccharides or other secondary metabolites.

In contrast to the original protocol, which is optimized for high-quality and high-integrity input DNA, this version provides a more realistic workflow for plant material that often yields partially degraded DNA. The main innovations are the incorporation of two sequential size-selection steps, freeze-and-squeeze^5^ gel recovery and a controlled two-step PCR enrichment strategy^6^ with intermediate bead-cleanup steps. In addition, the protocol simplifies restriction-ligation setup by using a common buffer compatible with all restriction enzymes, thereby avoiding the multiple buffer requirements of the original workflow and reducing handling complexity. The workflow also replaces EcoT22I with NsiI-HF, modernizing the protocol without altering its underlying enzymatic design. Together, these modifications improve recovery of fragments within the desired size range and reduce the carryover of residual primers and short DNA molecules prior to Illumina sequencing.

### Extraction and preparation of genomic DNA from plant tissue

**Timing: 2–4 h**

1. Collect plant material under as consistent conditions as possible to reduce biological variation and improve comparability in downstream epigenomic analyses.
  ***Note:*** For example, use the same developmental stage, tissue type, collection time and environmental conditions.
  a. Immediately freeze the material in liquid nitrogen and grind each sample using a mortar and pestle.
    ***Note:*** Alternatively, maintain the collected plant material on dry ice, pre-cool TissueLyser adapters with liquid nitrogen and homogenize the tissue using the TissueLyser at 25 Hz for 1 min. If visible leaf fragments remain, repeat as needed until a homogeneous powder is obtained, while maintaining the cold chain and avoiding sample thawing.
  b. Use the resulting powder to extract genomic DNA using DNeasy Plant Mini Kit (Qiagen), following the manufacturer’s instructions.
    ***Note:*** Other plant DNA extraction kits or established protocols, such as CTAB-based methods, may be used if they provide sufficient DNA quantity, purity and integrity.
2. Quantify DNA and assess genomic DNA purity.
  a. Load 2 μL of each DNA sample on a NanoDrop spectrophotometer.
  b. Record DNA concentration and A260/280 and A260/230 ratios.

**CRITICAL:** For the complete workflow, at least 300 ng total genomic DNA per biological sample is recommended, corresponding to 100 ng minimum per enzyme-specific restriction-ligation reaction. If DNA quantity is limiting, repeat or optimize DNA extraction.

***Note:*** Samples with low purity may affect downstream library preparation efficiency. Good-quality DNA is expected to show an A260/280 ratio close to 1.8. Values far from this range may indicate contamination. The A260/A230 ratio is informative but may be more variable due to secondary metabolites or salts.

3. Assess genomic DNA quality and integrity by agarose gel electrophoresis.
  a. Prepare a 1.2% (w/v) agarose gel.
  b. Load 50–100 ng of DNA per sample.
  c. Include a molecular weight marker (1 kb Plus DNA Ladder).
  d. Run the gel and evaluate DNA integrity.

**Figure 1:**
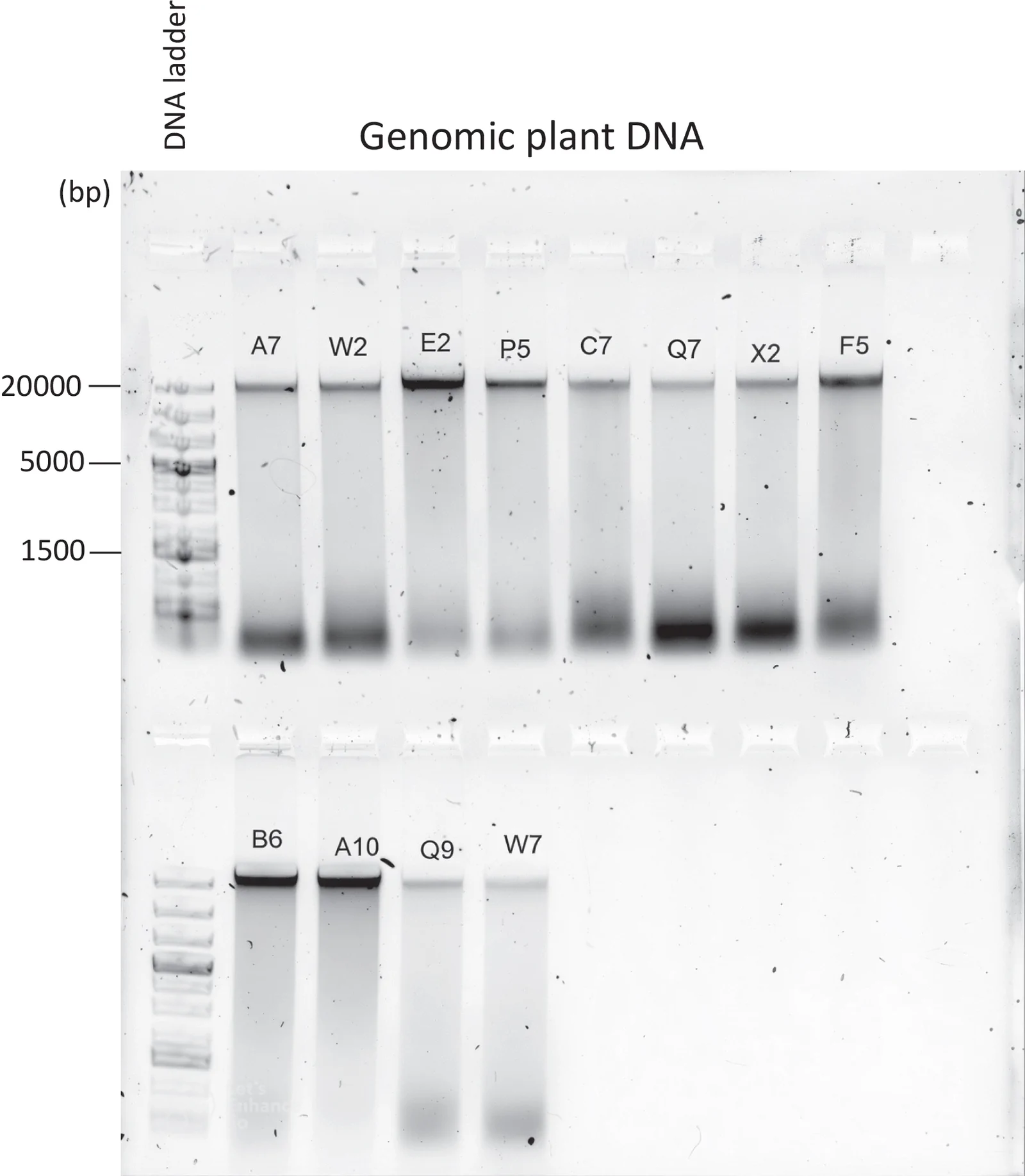
Agarose gel electrophoresis (1.2%) of genomic DNA from multiple samples High-molecular-weight bands are visible in all samples, although faint smearing suggests partial DNA degradation.

***Note:*** High-molecular-weight bands should be visible, although some faint smearing may be observed in partially degraded samples (Figure 1).

### Preparation of enzyme-specific barcoded adapters and indexed PCR primers

**Timing: 3–5 h**

4. Order the adapter set required for each library construction, including the specific TOP and BOT barcoded adapters for each methylation-sensitive restriction enzyme (AciI, NsiI-HF and PstI-HF), the adapters corresponding to the methylation-insensitive restriction enzyme MseI, and the indexed PCR primers (Table S1).

**CRITICAL:** Plan barcode assignment and index combinations before starting the restriction-ligation reactions to avoid sample misidentification during library preparation and sequencing.

***Note:*** This enzyme combination is recommended for the optimized workflow. Alternative enzymes may be used, but they would require redesigning the corresponding adapters and re-optimizing digestion-ligation conditions and buffer compatibility.

***Note:***Table S1 lists the oligonucleotide set used for MCSeEd library preparation. The barcode system was developed for epigenomic libraries consisting of 10 samples per library for subsequent sequencing by MCSeEd. For each methylation-sensitive restriction enzyme, 20 adapters are included (10 forward and 10 reverse). These adapters share a common sequence but differ in their unique seven-nucleotide barcode, highlighted in bold. Table S1 also includes the common MseI adapter, the common PCR primer (PCR1_MCSeEd) and 10 indexed PCR2 Illumina primers, each carrying a unique eight-base index sequence, also highlighted in bold.

5. Dissolve each single-stranded adapter oligonucleotide in ultra-pure water to a final concentration of 100 μM.

***Note:*** Oligonucleotides supplied pre-normalized to 100 μM in TE buffer (pH 8.0) may be compatible, but adapter annealing efficiency should be verified before use.

6. Mix each TOP oligonucleotide with its complementary BOT oligonucleotide in a 1:1 ratio to generate double-stranded adapters (final concentration of 50 μM).
7. Anneal the adapters using the temperature profile indicated in Table 1.

**Table 1:** Adapter annealing temperature profile

Steps	Temperature	Time	Cycles
Initial Denaturation	95°C	3 min	1
Cooling ramp	–	–	0.1°C/s
Annealing	70 °C	10 min	1
Cooling ramp	–	–	0.1°C/s
Final extension	20°C	20 min	1
Hold	10°C	forever
8. Verify adapter annealing by electrophoresis on a 4% agarose gel (Figure 2).

**Figure 2:**
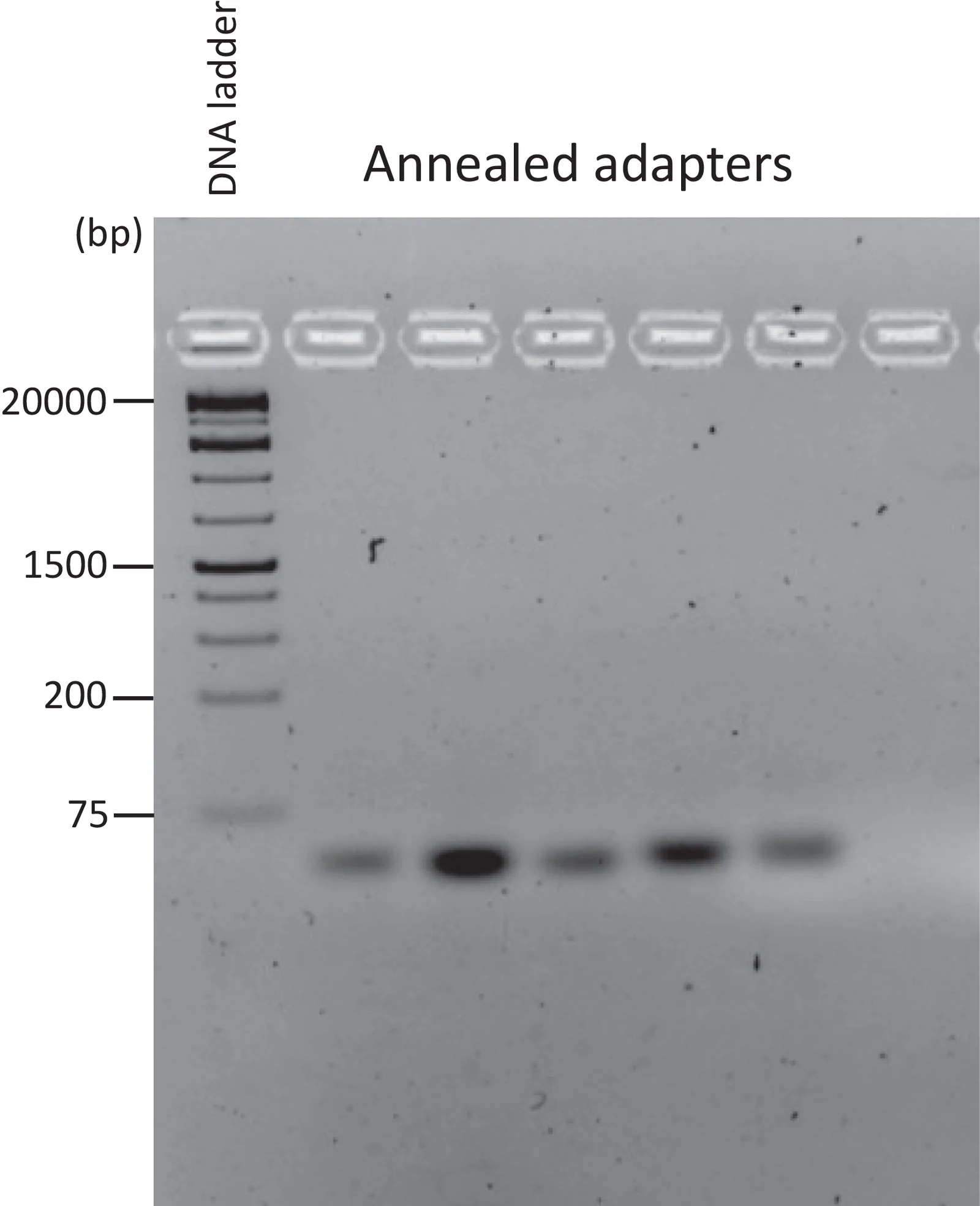
Agarose gel electrophoresis showing annealed adapter oligonucleotides Successful adapter annealing is indicated by clear, homogeneous bands corresponding to the expected double-stranded adapter size. Non-annealed single-stranded oligonucleotides can be loaded separately as an additional control; these are expected to migrate lower than the annealed adapter product.

### Preparation of restriction-ligation and purification reagents

**Timing: 30–45 min**

9. Prepare fresh 5× RL buffer as described in materials and equipment.
10. Assign the appropriate adapter combinations and index sequences to each sample before starting the restriction-ligation reactions.
11. Ensure that all restriction enzymes, ATP, T4 DNA ligase and reaction plates are available and maintained under the recommended storage and handling conditions until use.
12. Prepare fresh 70% (v/v) ethanol on the day of use.
13. Equilibrate AMPure beads to room temperature for 30 min before use.

***Note:*** Equivalent SPRI-based magnetic bead purification systems may be used, but bead-to-sample ratios, DNA recovery and fragment-size selection behavior should be validated before routine use.

14. Confirm that a magnetic rack or magnetic plate is available and functioning correctly.

### Preparation for PCRs, electrophoresis, and final quality control

**Timing: 45–60 min**

15. Prepare 1.2% (w/v) agarose gels for genomic DNA quality assessment and size-selection steps.
16. Prepare a 1.5% (w/v) agarose gel for evaluation of PCR enrichment and verification of primer removal after purification.
17. Prepare loading dye, an appropriate DNA ladder (1 kb Plus DNA Ladder), and a nucleic acid stain before starting electrophoresis.

***Note:*** This protocol was performed using SYBR Safe DNA Gel Stain. Equivalent nucleic acid stains may be used according to manufacturer’s recommendations.

18. Confirm the availability of Phusion High-Fidelity DNA polymerase, PCR primers and PCR indices required for the two enrichment PCR steps.

***Note:*** Other high-fidelity DNA polymerases may be compatible but should be validated before routine use.

19. Ensure that Qubit HS reagents and standards are at room temperature and available for final DNA quantification.

## Key resources table

**Table:** 

REAGENT or RESOURCE	SOURCE	IDENTIFIER
**Biological samples**		

Leaf-tissue samples from *Nardus stricta* used for genomic DNA extraction	This study	N/A

**Chemicals, peptides, and recombinant proteins**		

AciI restriction enzyme	New England Biolabs	Cat#R0551S
NsiI-HF® restriction enzyme	New England Biolabs	Cat#R3127S
PstI-HF® restriction enzyme	New England Biolabs	Cat#R3140S
MseI restriction enzyme	New England Biolabs	Cat#R0525S
T4 DNA ligase	Thermo Fisher Scientific	Cat#EL0011
rCutSmart Buffer	New England Biolabs	Cat#B6004S
Agarose E GEN	Labkem/Labbox	Cat#AGAR-E00-500
DNA Gel Loading Dye, 6×	Thermo Scientific/Thermo Fisher Scientific	Cat#R0611
SYBR Safe DNA Gel Stain	Thermo Fisher Scientific/Invitrogen	Cat#S33102
ATP solution	New England Biolabs	Cat#P0756S
Dithiothreitol (DTT)	Thermo Fisher Scientific	Cat#J15397.03
PEG8000	Thermo Fisher Scientific	Cat#043443.22
Magnesium chloride (MgCl_2_)	Thermo Fisher Scientific	Cat#223211000

**Critical commercial assays**		

DNeasy Plant Kit	Qiagen	Cat#69106
AMPure XP beads	Beckman Coulter	Cat#A63881
Phusion High-Fidelity DNA Polymerase	New England Biolabs	Cat#M0530S
O’GeneRuler 1 kb DNA Ladder Ready-to-Use	Thermo Scientific/Thermo Fisher	Cat#11551615
Qubit dsDNA HS Assay Kit	Thermo Fisher Scientific	Cat#Q32851
TapeStation High Sensitivity D1000 ScreenTape	Agilent Technologies	Cat#5067-5584
TapeStation High Sensitivity D1000 Reagents	Agilent Technologies	Cat#5067-5585

**Oligonucleotides**		

Barcoded adapters for PstI-HF, AciI and NsiI-HF	This study	See Table S1
Common MseI adapter	This study	See Table S1
PCR1_MCSeEd primer	This study	See Table S1
PCR2_Index primers	This study	See Table S1

**Other**		

TissueLyser II	Qiagen	Cat#85300
Nanodrop spectrophotometer	Thermo Fisher Scientific	Cat#ND-ONE-W
T100 Thermal cycler	Bio-Rad	Cat#1861096
Magnetic rack or magnetic plate	New England Biolabs	Cat#S1515S
Wide Mini ReadySub-Cell GT Horizontal Electrophoresis System, for precast gels	Bio-Rad Laboratories	Cat#1704489
Gel Doc EZ System	Bio-Rad Laboratories	Cat#1708270
Qubit 4 Fluorometer	Thermo Fisher Scientific	Cat#Q33238
4200 TapeStation System	Agilent Technologies	Cat#G2991BA

## Materials and equipment

**Table:** 5× RL buffer (rCutSmart-based)

Reagent	Final concentration	Amount
10× rCutSmart buffer	5×	500 μL
DDT (1 M)	25 mM	25 μL
Ultra-pure water	N/A	475 μL
**Total**	**N/A**	**1 mL**

Store at −20°C for extended periods. Thaw completely and mix well before use.

**CRITICAL:** Dithiothreitol (DTT) is harmful if inhaled, swallowed, or absorbed through the skin and may cause irritation to the eyes, skin, and respiratory tract. Prepare the buffer in a well-ventilated area and wear appropriate personal protective equipment, including gloves, lab coat and eye protection.

**Table:** PEG solution

Reagent	Final concentration	Amount
PEG8000	30% (w/v)	0.3 g
MgCl_2_ (1 M)	30 mM	30 μL
Ultra-pure water	N/A	To 1 mL
**Total**	**N/A**	**1 mL**

Prepare fresh on the day of use. Do not store.

***Note:*** Add MgCl_2_ to approximately 600 μL of ultra-pure water before adding PEG8000 and then adjust to the final volume of 1 mL after the PEG8000 has fully dissolved.

**CRITICAL:** PEG8000 may require time to dissolve completely. Vortex thoroughly and do not use the solution until it is fully clear. Filter the solution through a 0.2 μm filter before use.

## Step-by-step method details

This protocol is typically completed over 2 days. **Day 1** includes restriction digestion and adapter ligation of genomic DNA, pooling and initial purification, and the first size-selection and freeze-and-squeeze recovery steps. **Day 2** includes the second size-selection step, recovery of the selected library fraction, sequential enrichment PCR and cleanup, and final library quantification and quality assessment.

### Restriction digestion and adapter ligation of genomic DNA

**Timing: 4.5–5 h**

This step generates the initial MCSeEd library molecules by digesting genomic DNA with three methylation-sensitive enzymes (AciI, NsiI-HF and PstI-HF) in combination with MseI, followed by adapter ligation in a one-step reaction. Each genomic DNA sample is divided into three subsamples, each processed with a different methylation-sensitive enzyme (Table 2).

1. Divide the genomic DNA from each sample into three equal subsamples.

***Note:*** From this point onward, low-bind DNA tubes, such as DNA LoBind Tubes or equivalent products, may be considered to minimize DNA loss during the multiple cleanup, size-selection and transfer steps.

2. Assign the appropriate barcoded adapters and PCR indices to each sample according to the experimental design (Table S1).
3. Prepare the one-step restriction-ligation using the reagent composition indicated in Table 3.

**Table 3:** Reaction composition and volumes for the one-step restriction-ligation reactions

Reagent	Amount
5× RL buffer (rCutSmart)	10 μL
Methylation-sensitive restriction enzyme AciI and NsiI-HF® (10 U/μL)	0.5 μL
PstI-HF® restriction enzyme (20 U/μL)	0.25 μL
MseI restriction enzyme (10 U/μL)	0.5 μL
Barcoded adapter (50 μM)	2 μL
MseI adapter (50 μM)	2 μL
ATP (10 mM)	1 μL
T4 DNA ligase (5 U/μL)	0.2 μL
Ultra-pure water	To 50 μL
gDNA	100−200 ng per subsample
4. Incubate at 37°C for 4 h.
5. Meanwhile, prepare fresh PEG solution as described in materials and equipment.

**Table 2:** Example distribution of a single genomic DNA sample into three enzyme-specific subsamples for restriction-ligation reactions, each containing one methylation-sensitive restriction enzyme and its corresponding barcoded adapter, together with the methylation-insensitive enzyme MseI and the MseI adapter

Sample name	Subsample	Methylation- sensitive enzyme	Common enzyme	Barcoded adapter	Common adapter
Sample 1	1A	AciI	MseI	AciI barcode	MseI adapter
	1B	NsiI-HF	MseI	NsiI-HF barcode	MseI adapter
	1C	PstI-HF	MseI	PstI-HF barcode	MseI adapter

### Pooling of ligated DNA and initial purification

**Timing: 1 h**

This step pools ligated samples according to their common PCR index and removes excess reaction components before size selection. A PEG precipitation is followed by a 1.1× AMPure cleanup to enrich fragments above 250 bp.

6. Combine ligated DNA samples to create pools of individuals to be amplified with a common PCR index.
7. Purify each pool with PEG solution.
  a. Add 0.5× volume of PEG solution to each pooled sample.
  b. Mix thoroughly by pipetting up and down at least 20 times.
  c. Centrifuge at 10,000 × *g* for 20 min at room temperature.
  d. Quickly remove and discard the supernatant.
  e. Resuspend the DNA pellet in 30 μL of ultra-pure water.

**CRITICAL:** Resuspend the pellet gently and avoid introducing bubbles.

8. Perform a 1.1× AMPure cleanup to remove fragments shorter than 250 bp.
  a. Vortex the AMPure bead stock thoroughly to ensure complete resuspension and add 33 μL of AMPure beads to 30 μL of pooled library sample.
  b. Mix by pipetting up and down for at least 10 times and incubate for 5 min at room temperature.
  c. Place the tubes on a magnetic rack for at least 5 min, or until the beads have fully separated from the solution.
  d. Keeping the microcentrifuge tubes on the magnetic rack, carefully remove and discard supernatant.
  e. With the microcentrifuge tubes still on the magnet, add 100 μL of 70% ethanol (v/v) to each sample, incubate for 30 s at room temperature and remove the ethanol.
  f. Repeat the ethanol wash step two additional times, for a total of three washes.
  g. After the third wash, remove as much ethanol as possible, and let beads air-dry until ethanol has completely evaporated.
    **CRITICAL:** Do not over-dry the beads to the point of cracking.
    ***Note:*** To facilitate removal of residual ethanol after the third wash, the tubes may be briefly centrifuged off the magnetic rack. Then return the tubes to the magnetic rack and carefully remove any ethanol collected at the bottom of the tube.
  h. Remove samples from the magnetic plate and add 21 μL of ultra-pure water to each sample.
  i. Mix by pipetting at least 25 times and incubate for 5 min at room temperature.
  j. Place the samples back on the magnetic plate and incubate until the beads have pelleted and the supernatant is clear.
  k. Transfer 20 μL of the supernatant to a new tube.
    **Pause point:** Samples can be stored overnight at −20°C overnight before proceeding to the first size-selection step. If longer storage is required, elution in low-EDTA buffer such as 0.1× TE may be considered to improve DNA stability.

### First size selection and recovery of target fragments

**Timing: 2.5–3 h**

This step enriches the library for DNA fragments within the range required for Illumina sequencing. Size selection is followed by freeze-and-squeeze recovery and 0.8× AMPure cleanup.

**Figure 3:**
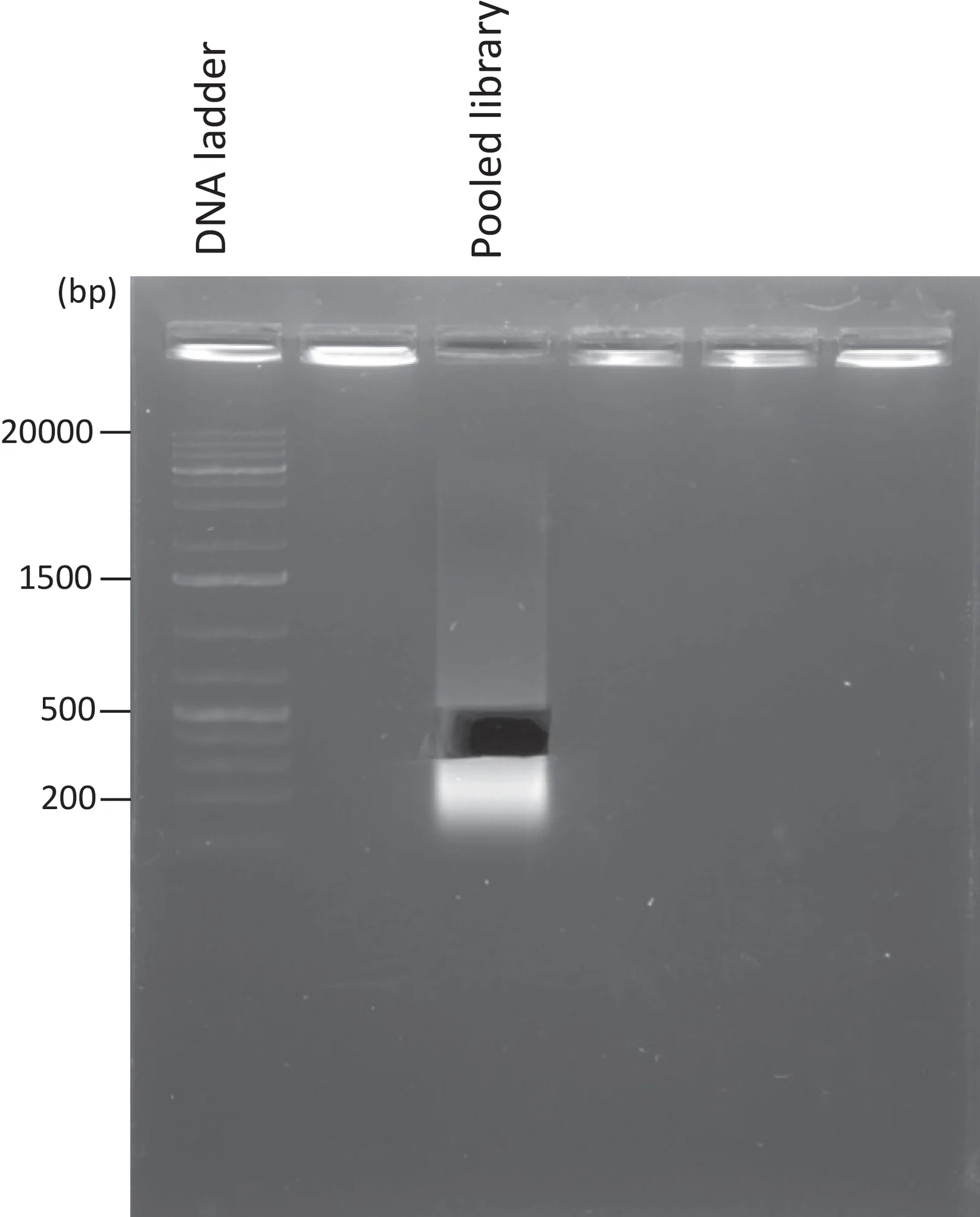
First size-selection step showing the excised gel region corresponding to DNA fragments between 250 and 600 bp To minimize carryover of small fragments during subsequent library preparation, it is preferable to excise slightly above 250 bp.

9. Prepare a 1.2% (w/v) agarose gel and add 5 μL of nucleic acid stain.
10. Add 4 μL of loading dye to each sample and load the samples onto the gel.
11. Include an appropriate molecular weight marker (1 kb DNA ladder).
12. Run the gel at 50 V for 90 min.
13. Excise DNA fragments within the 250–600 bp range (Figure 3).

**CRITICAL:** Minimize exposure to UV light during gel excision, as UV irradiation can damage DNA.

***Note:*** A blue-light transilluminator, such as Safe Imager™ or an equivalent system, could be used to minimize DNA damage during gel excision.

14. Recover the excised DNA fragments using the freeze-and-squeeze method.
  ***Note:*** During protocol optimization, the GeneJET Gel Extraction Kit (Thermo Scientific, Cat. No. K0691) was tested as an alternative gel-recovery method. However, the freeze-and-squeeze method was retained because it provided better DNA recovery in our hands.
  Other commercial gel extraction kits remain possible alternatives but should be validated before routine use.
  a. Cut the end of a pipette tip and place it inside a 1.5 mL microcentrifuge tube.
  b. Transfer the excised gel fragment into the modified pipette tip.
  c. Close the tube and freeze at −80°C for 5 min.
  d. Centrifuge immediately for 3 min at maximum speed.
15. Measure the recovered volume in each tube.
16. Perform a 0.8× AMPure cleanup using the measured recovered volume.
  a. Vortex the AMPure bead stock thoroughly to ensure complete resuspension and add a volume of AMPure beads equivalent to 0.8× the measured sample volume.
  b. Mix by pipetting up and down for at least 10 times and incubate for 5 min at room temperature.
  c. Place the tubes on a magnetic rack for at least 5 min, or until the beads have fully separated from the solution.
  d. Keeping the microcentrifuge tubes on the magnetic rack, carefully remove and discard supernatant.
  e. With the tubes still on the magnetic rack, wash the beads by adding 200 μL of 70% ethanol (v/v) to each sample and incubating for 30 s at room temperature and remove the ethanol.
    ***Note:*** If the initial total recovered volume is less than 100 μL, use 100 μL of 70% ethanol (v/v) instead.
  f. Repeat the ethanol wash step two additional times, for a total of three washes.
  g. After the third wash, remove as much ethanol as possible, and let beads air-dry until residual ethanol has evaporated.
    **CRITICAL:** Do not over-dry the beads to the point of cracking.
    ***Note:*** To facilitate removal of residual ethanol after the third wash, the tubes may be briefly centrifuged off the magnetic rack. Then return the tubes to the magnetic rack and carefully remove any ethanol collected at the bottom of the tube.
  h. Remove samples from the magnetic plate and add 31 μL of ultra-pure water to each sample.
  i. Mix by pipetting at least 25 times and incubate for 5 min at room temperature.
  j. Place the samples back on the magnetic plate and incubate until the beads have pelleted and the supernatant is clear.
  k. Transfer 30 μL of the supernatant to a new tube.
    **Pause point**: Samples can be stored overnight at −20°C before proceeding to the second size-selection step. If longer storage is required, elution in low-EDTA buffer such as 0.1× TE may be considered to improve DNA stability.

### Second size selection and recovery of the selected library fraction

**Timing: 2.5–3 h**

This step further reduces carryover of short DNA fragments that may be preferentially amplified during enrichment PCR. The procedure is identical to the first size-selection step but is applied to the purified library fraction obtained after the 0.8× AMPure cleanup.

17. Perform a second size selection by repeating steps 9–14 using the purified DNA obtained in step 16.

**Figure 4:**
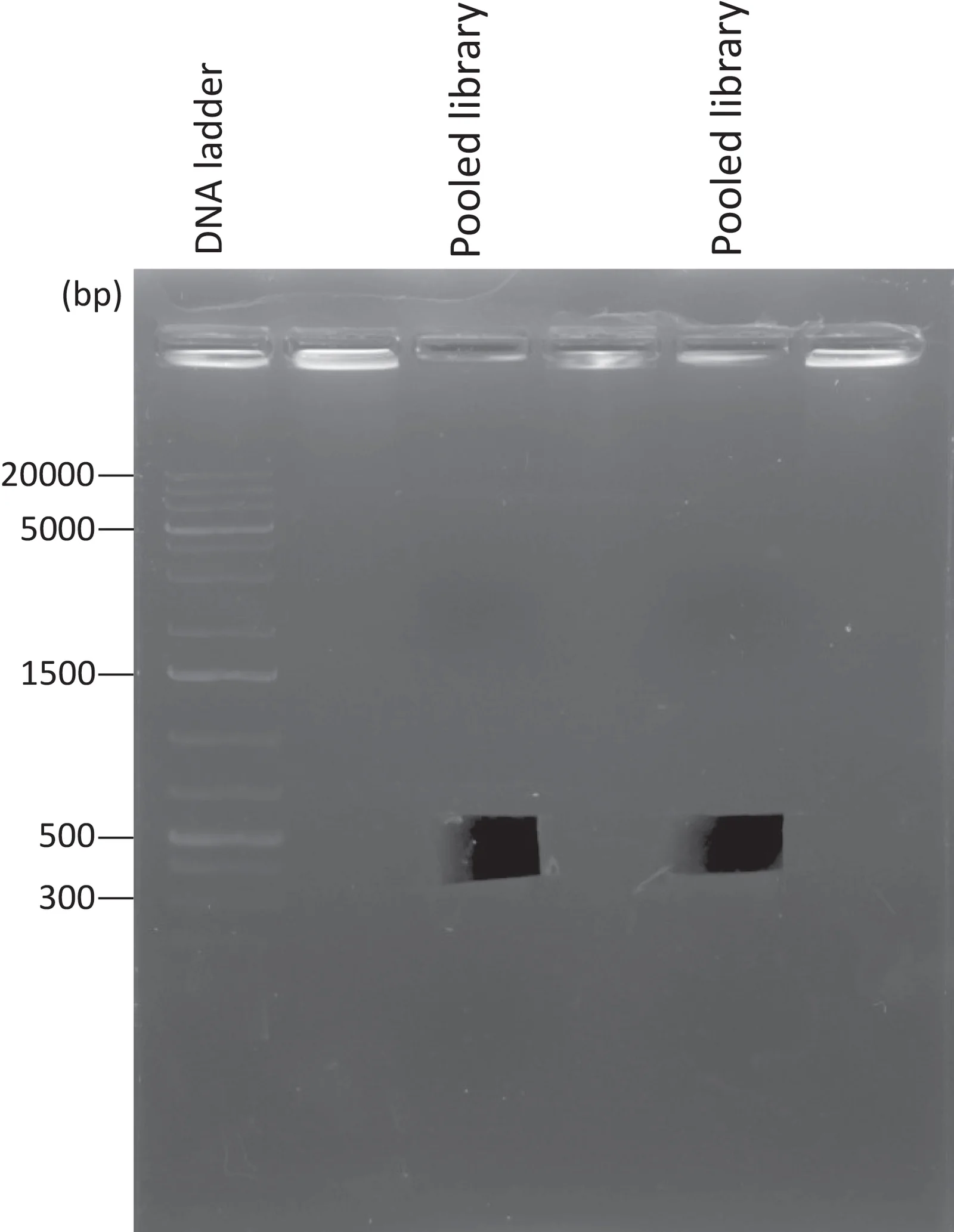
Second size-selection step showing the excised gel region corresponding to DNA fragments between 250 and 600 bp To minimize carryover of small fragments during subsequent library preparation, it is preferable to excise slightly above 250 bp. The DNA smear is less pronounced that in the first size-selection step, while sufficient DNA material is still retained for downstream enrichment PCRs.

***Note:*** At this stage, the DNA smear is typically less pronounced than during the first size selection, but sufficient DNA material is retained for downstream enrichment PCR (Figure 4).

### Sequential enrichment PCR and intermediate cleanup

**Timing: 3–4 h**

This step selectively enriches fragments carrying both the common MseI adapter and the barcoded adapter and incorporates the index sequence required for sequencing. Two sequential PCRs are performed, separated by a 1× AMPure cleanup to remove residual primers and reaction contaminants.

18. Perform the first enrichment PCR using the reaction composition shown in Table 4 and the cycling conditions shown in Table 5.

**Table 4:** Composition of the enrichment PCR reaction

Reagent	Amount
DNA template	15 ng
5× Phusion HF buffer	10 μL
5 μM dNTPs	2 μL
10 mM PCR1_MCSeEd	1 μL
10 mM PCR2_Index_X	1 μL
Phusion High-Fidelity DNA polymerase	1 U
Nuclease-free water	To 50 μL

**Table 5:** Cycling conditions for first and the second enrichment PCRs

Steps	Temperature	Time	Cycles
Initial Denaturation	98°C	2 min	1
Denaturation	98°C	20 sec	**7 cycles** for first enrichment PCR and **14 cycles** for second enrichment PCR
Annealing	56°C	30 sec	
Extension	72°C	20 sec	
Final extension	72°C	5 min	1
Hold	10°C	forever

***Note:*** Use 15 ng of input DNA when the concentration can be reliably determined (e.g., within the accurate detection range of the quantification method). However, at this stage, DNA concentrations are often too low for accurate quantification. In such cases, add 28 μL of template DNA directly to the PCR reaction. For this reason, Qubit quantification is recommended after the final purification step rather than before PCR enrichment.

19. Run the first enrichment PCR for 7 cycles.
20. Purify the amplified libraries with a 1× AMPure cleanup.
  a. Vortex the AMPure bead stock thoroughly to ensure complete resuspension and add 50 μL of AMPure beads to 50 μL of pooled library sample.
  b. Mix by pipetting up and down for at least 10 times and incubate for 5 min at room temperature.
  c. Place the tubes on a magnetic rack for at least 5 min, or until the beads have fully separated from the solution.
  d. Keeping the microcentrifuge tubes on the magnetic rack, carefully remove and discard supernatant.
  e. With the microcentrifuge tubes still on the magnet, add 100 μL of 70% ethanol (v/v) to each sample, incubate for 30 s at room temperature and remove the ethanol.
  f. Repeat the ethanol wash step two additional times, for a total of three washes.
  g. After the third wash, remove as much ethanol as possible, and let beads air-dry until ethanol has completely evaporated.
    **CRITICAL:** Do not over-dry the beads to the point of cracking.
    ***Note:*** To facilitate removal of residual ethanol after the third wash, the tubes may be briefly centrifuged off the magnetic rack. Then return the tubes to the magnetic rack and carefully remove any ethanol collected at the bottom of the tube.
  h. Remove samples from the magnetic plate and add 31 μL of ultra-pure water to each sample.
  i. Mix by pipetting at least 25 times and incubate for 5 min at room temperature.
  j. Place the samples back on the magnetic plate and incubate until the beads have pelleted and the supernatant is clear.
  k. Transfer 30 μL of the supernatant to a new tube.
21. Perform the second enrichment PCR using the same reaction composition shown in Table 4 and the same cycling conditions shown in Table 5 but increase amplification to 14 cycles.
22. Run 5 μL of each enriched library on a 1.5% (w/v) agarose gel to assess library enrichment (Figure 5).

**Figure 5:**
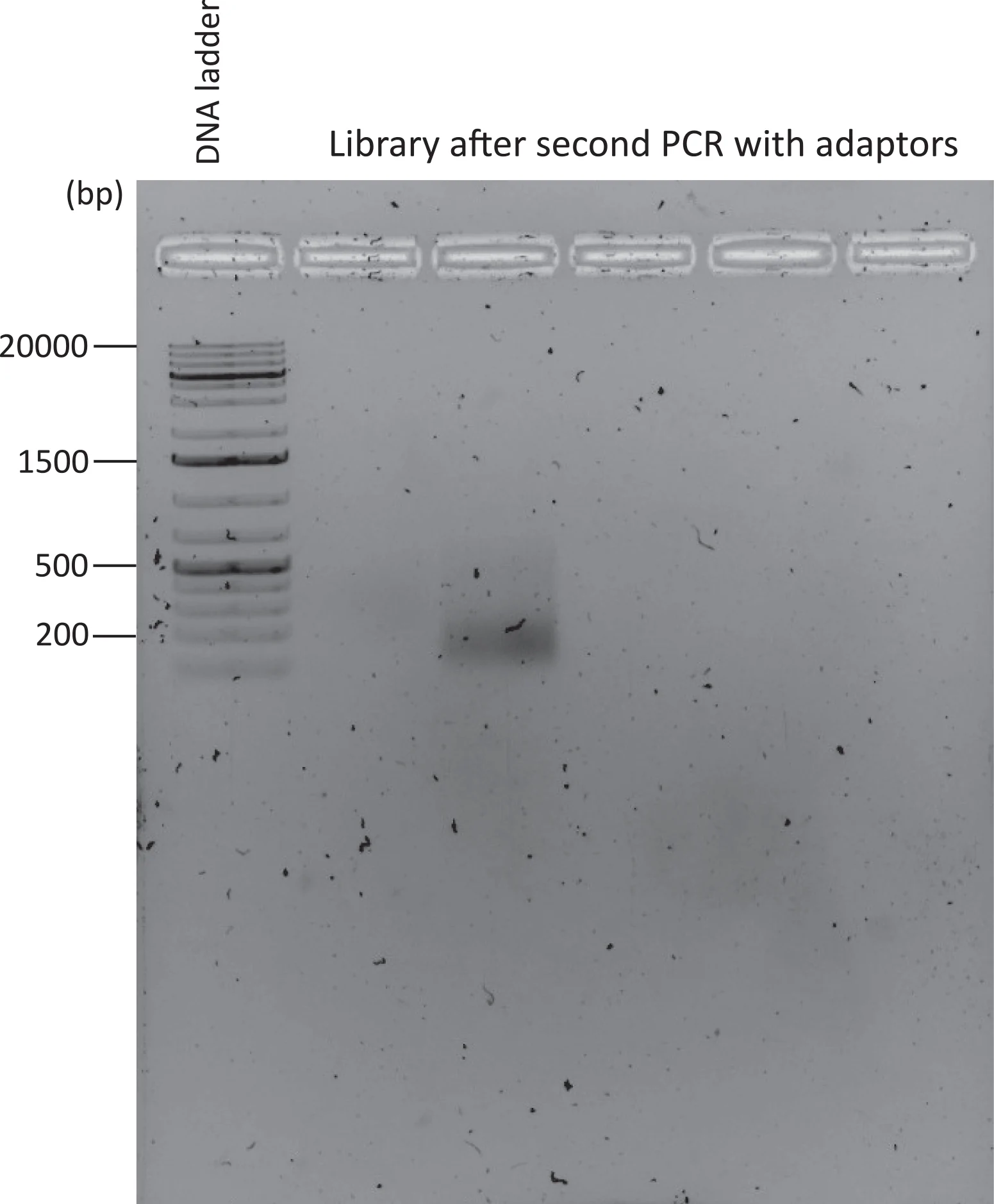
Electrophoretic profile of the library after the second enrichment PCR prior to the final bead purification step The low-molecular-weight band corresponds to residual PCR primers, which are removed during the subsequent purification step.
23. Purify the amplified libraries with a second 1× AMPure cleanup.
  a. Vortex the AMPure bead stock thoroughly to ensure complete resuspension and add 45 μL of AMPure beads to 45 μL of pooled library sample.
  b. Repeat the rest of sub-steps explained in step 20.

**CRITICAL:** Confirm sequencing provider requirements before starting final quality-control assays. The final elution volume is 30 μL, if ≥25 μL of library pool is required for submission, reserve sufficient volume before performing subsequent QC steps. Agarose gel assessment is useful to obtain a general overview of library quality and residual primer carryover, whereas Qubit quantification provides high accurate DNA concentration. When library volume is limiting, users may prioritize TapeStation analysis, as this system requires low input volume and provide both fragment-size distribution and an estimated library concentration.

**Figure 6:**
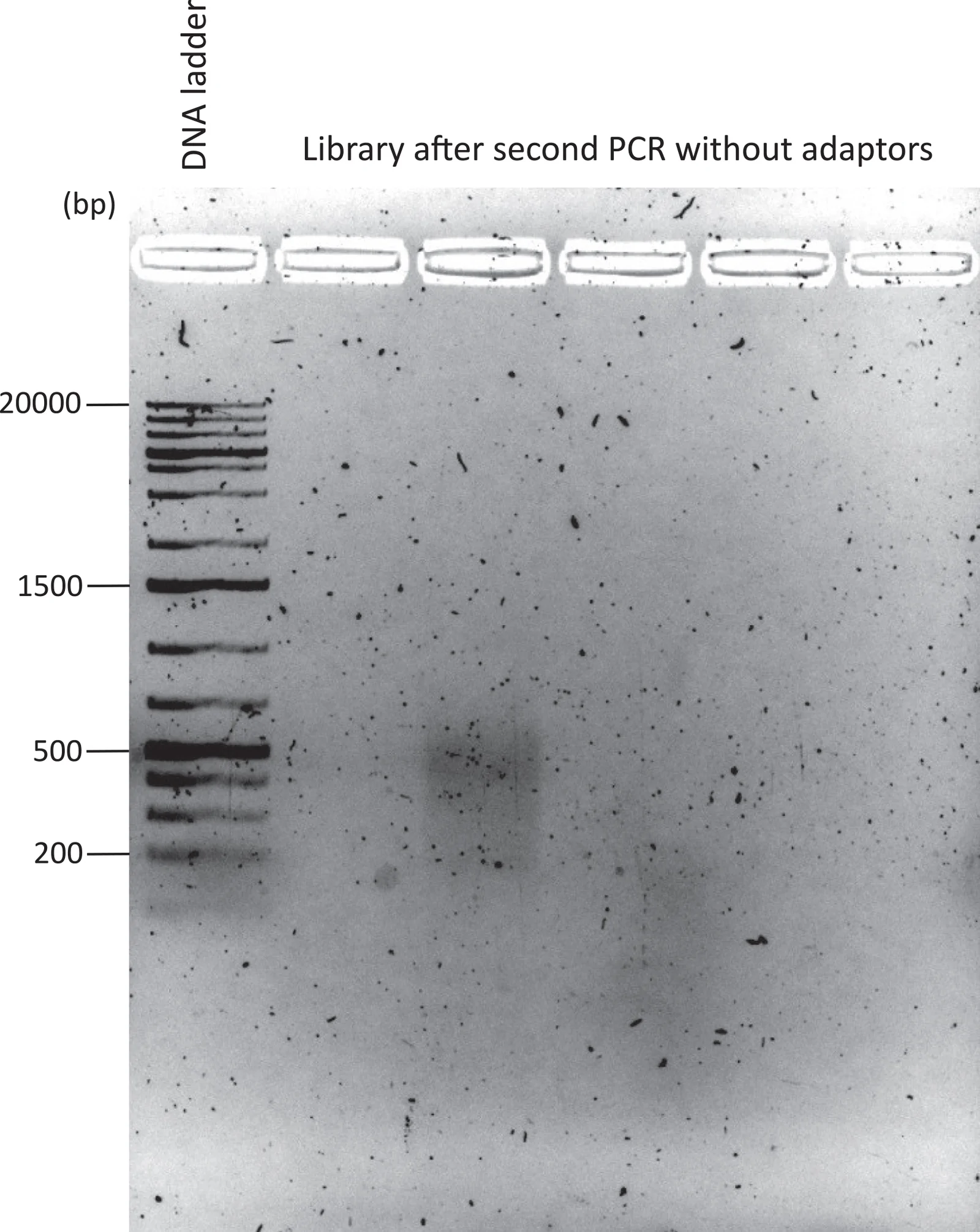
Electrophoretic profile of the library after the final bead purification step The low-molecular-weight band corresponding to residual PCR primers is no longer present.

24. Run 5 μL of each enriched library on a 1.5% agarose gel (w/v) to confirm the absence of residual PCR primers (Figure 6).

**Pause point:** Samples can be stored overnight at −20°C overnight before final library quantification and TapeStation analysis. If longer storage is required, elution in low-EDTA buffer such as 0.1× TE may be considered to improve DNA stability.

### Final library quantification and quality assessment

**Timing: 20–30 min**

This step determines the final DNA concentration of the purified libraries and evaluates fragment-size distribution prior to sequencing. Final quality control is performed using Qubit and TapeStation analysis.

**Figure 7:**
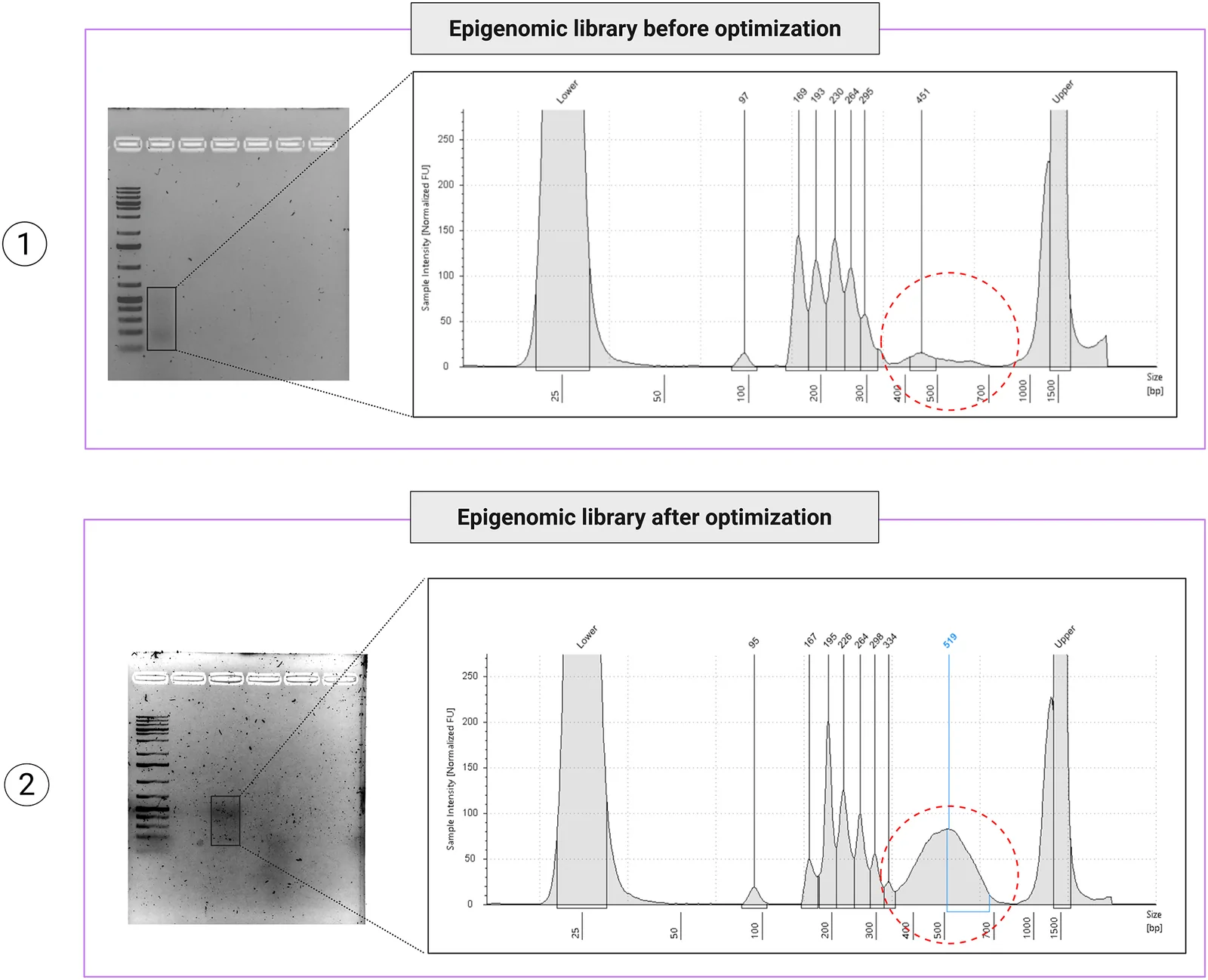
TapeStation analysis of the prepared libraries before and after protocol optimization (1) Library profile obtained prior to optimization using the original MCSeEd protocol described by Di Marsico et al., showing prominent low-molecular-weight peaks and minimal DNA within the target 400–600 bp size range. (2) Library profile obtained after applying the optimized workflow, showing that although low-molecular-weight peaks are still present, substantial DNA is recovered within the target 400–600 bp range.

***Note:*** Other equivalent fragment-analysis systems, such as 2100 Bioanalyzer, may also be used for final library control.

25. Allow the Qubit HS reagents, standards, and DNA samples to equilibrate to room temperature before preparing the working solution.
26. Prepare the Qubit working solution by mixing 199 μL of HS buffer with 1 μL of HS reagent per sample.
27. Mix thoroughly by vortexing.
28. Dispense 190 μL of the working solution into each Qubit tube labeled S1 (Standard 1) and S2 (Standard 2).
29. Add 10 μL of S1 and S2 to their respective tubes.
30. Dispense 198 μL of the working solution into a Qubit tube for each DNA sample.
31. Add 2 μL of purified DNA to each sample, for a final volume of 200 μL.
32. Vortex each tube for 2–3 s and incubate at room temperature for 2 min.
33. Calibrate the instrument using the two standards.
34. Measure each sample.
35. Assess the final library profile using the TapeStation system according to the manufacturer’s instructions and standard procedures for the technique (Figure 7).

## Expected outcomes

The protocol is expected to generate MCSeEd libraries suitable for Illumina sequencing from plant genomic DNA of suboptimal quality or integrity. After DNA extraction, genomic DNA typically shows high-molecular-weight bands with some degree of smearing, consistent with partial degradation commonly observed in plant samples (Figure 1). Following adapter ligation, purification and double size selection, the library should be enriched for fragments within the desired size range, while carryover of low-molecular-weight fragments is progressively reduced. After the two enrichment PCR steps and the final bead purification, residual PCR primers should no longer be visible on agarose gels (Figure 6). Final library quality assessment by TapeStation is expected to show a broad fragment-size distribution compatible with Illumina sequencing, although minor low-molecular-weight peaks may still be present (Figure 7).

Final library yield may vary depending on starting DNA quality and DNA loss during cleanup, size-selection, and transfer steps. In the representative optimized library shown in Figure 7, the final concentration was 1.61 ng/μL in a 30 μL elution volume, corresponding to approximately 48 ng of total library DNA. The library yield and TapeStation profiles were considered sufficient by the sequencing facility, which required ≥1.5 nM library pool and ≥25 μL library pool for a whole Illumina lane with 150-bp reads.

Overall, these pre-sequencing quality-control results indicate that the optimized workflow can generate MCSeEd libraries suitable for Illumina sequencing. Sequencing-level quality-control metrics should subsequently be assessed to evaluate downstream library performance, as discussed in the limitations section. As a reference for possible MCSeEd sequencing outputs, Marconi et al.^7^ reported a mean of 7.8 million 150-bp reads per library, with 98% of reads passing quality control and aligning to the reference genome. They also reported that 48.49% of total reads mapped uniquely and identified 992,320 cytosine-containing loci. These values should be considered reference expectations for MCSeEd-type libraries rather than fixed thresholds for the optimized workflow described here.

## Limitations

Although this protocol improves MCSeEd library preparation from plant genomic DNA of suboptimal quality or integrity, the quality of the starting DNA remains a major determinant of the final outcome. Highly degraded DNA may reduce library yield, alter fragment-size distribution and compromise downstream sequencing performance.

The protocol includes multiple purification and size-selection steps, which improve fragment recovery and reduce carryover of short fragments but also increase sample handling time and the risk of DNA loss. The two-step PCR enrichment strategy improves amplification of target fragments yet may still introduce amplification bias or favor residual low-molecular-weight molecules if size selection and cleanup are not performed carefully. Mechanical and technical factors, including incomplete PEG8000 dissolution, inaccurate gel excision, excessive UV exposure during band recovery, over-drying of magnetic beads or incomplete removal of ethanol may negatively affect library quality and reproducibility. As this protocol was optimized for plant material yielding partially degraded genomic DNA, its performance may vary across species, tissue types and extraction conditions, where additional optimization may be required for other biological systems.

Sequencing-based quality-control metrics were not evaluated as part of the present protocol optimization. Therefore, downstream library performance metrics, including mapping efficiency, duplication rate, fraction of usable reads, methylation-site coverage, context-specific coverage in CG, CHG and CHH sequence contexts and the number or proportion of informative restriction fragments contexts should be assessed after sequencing.

## Troubleshooting

### Problem 1

Low DNA yield or extensive degradation after extraction (related to before you begin, step 3).

Plant samples may yield low amounts of genomic DNA or highly degraded DNA because of rigid tissues, fibrous material and co-extracted contaminants. Excessive degradation at this stage may reduce library yield and compromise subsequent digestion, ligation and enrichment steps.

### Potential solution

**Figure 8:**
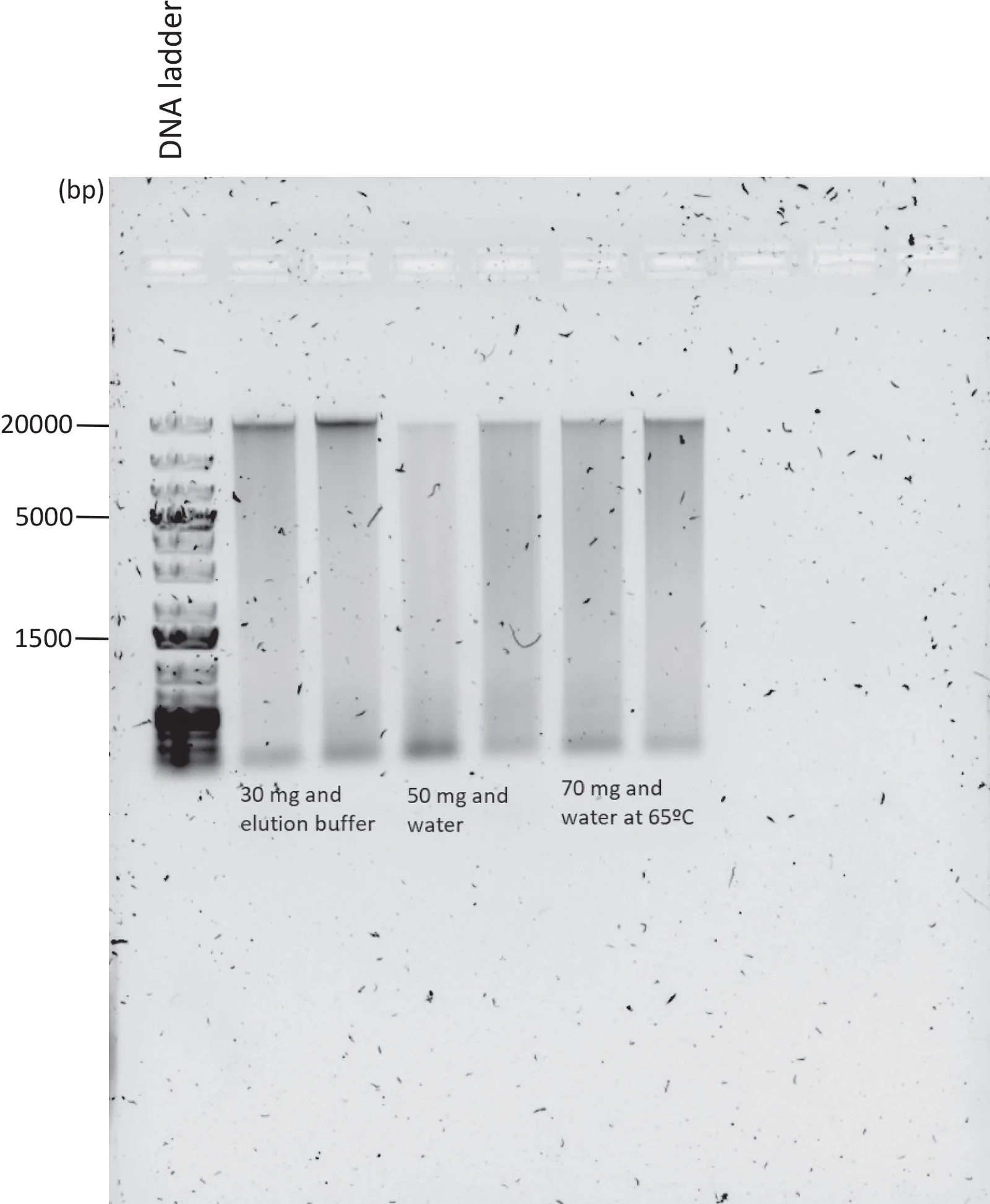
Comparison of genomic DNA extraction outcomes obtained under different elution conditions Improved DNA quality and integrity were observed when using a lower amount of starting plant material (30 mg) and eluting in elution buffer, compared with extractions performed using 50 mg or 70 mg of starting material and final elution in water at room temperature or water preheated to 65°C.
• If degradation remains severe, reduce input tissue quantity, handling time and optimize tissue disruption and extraction conditions for the target species.

• Process plant material immediately after collection and freeze it in liquid nitrogen or keep it on dry ice to minimize degradation.
• Grind tissue while keeping it frozen and reduce it to a fine powder to improve lysis efficiency and minimize the presence of larger tissue fragments.
• Elute DNA in elution buffer rather than ultra-pure water to improve DNA integrity (Figure 8).

### Problem 2

Incomplete adapter annealing or poor adapter performance (related to before you begin, step 8).

Improper annealing of TOP and BOT oligonucleotides may reduce ligation efficiency and lead to poor library recovery in later steps.

### Potential solution

• Confirm that each oligonucleotide has been resuspended to the correct concentration before annealing.
• Verify that complementary TOP and BOT oligonucleotides are mixed in the correct 1:1 ratio.
• Avoid repeated free-thaw cycles by preparing aliquots of annealed adapters.

### Problem 3

Low recovery after restriction-ligation and PEG purification (related to step-by-step method details, steps 3–7).

Insufficient digestion-ligation efficiency or poor pellet recovery after PEG precipitation may reduce the amount of DNA for the first AMPure purification.

### Potential solution

• Prepare the restriction-ligation carefully according to the reaction table and verify that all enzymes, ATP, and ligase are active and properly stored.
• Use the same 5× RL buffer composition consistently for all enzyme combinations.
• Prepare the PEG solution fresh and ensure that the PEG8000 is fully dissolved before use.
• After centrifugation, remove the supernatant quickly but carefully to avoid losing the pellet.
• Resuspend the pellet gently and completely in ultra-pure water before continuing.

### Problem 4

Loss of DNA during AMPure bead purification (related to step-by-step method details, steps 8, 16, 20 and 23).

Magnetic bead purification may result in low recovery if beads are not fully resuspended, if the pellet is disturbed during ethanol removal or if beads are over-dried before elution.

### Potential solution

• Vortex the room-temperature AMPure bead stock before each use.
• Allow sufficient time on the magnetic rack until the supernatant is completely clear before removing it.
• Remove ethanol carefully without the bead pellet.
• After the final ethanol wash, briefly centrifuge the tubes off the magnetic rack if needed, then return them to the magnetic rack and remove residual ethanol carefully.
• Do not over-dry the beads to the point of cracking, as this can reduce elution efficiency.

### Problem 5

Poor fragment recovery or incorrect size selection after gel excision (related to step-by-step method details, steps 13–14 and 17).

Magnetic bead purification may result in low recovery if beads are not fully resuspended, if the pellet is disturbed during ethanol removal or if beads are over-dried before elution.

### Potential solution

• Excise fragments within the 250–600 bp range, it is recommended to cut slightly above 250 bp to reduce carryover of shorter fragments.
• Minimize UV exposure during gel excision.
• Handle gel slices carefully during freeze-and-squeeze recovery to avoid sample loss.

### Problem 6

Weak amplification or absence of PCR product after enrichment PCR (related to step-by-step method details, steps 18–22).

Low DNA input, incomplete adapter ligation or poor library recovery in earlier cleanup steps may lead to weak or absent amplification during enrichment PCR.

### Potential solution

• Use 15 ng of input DNA when the concentration can be measured reliably.
• If DNA concentration is too low to quantify accurately at this stage, use 28 μL of template DNA as recommended in the protocol.
• Confirm that PCR primers, Phusion High-Fidelity DNA polymerase and cycling conditions are correct before repeating the reaction.
• Avoid introducing contaminants during the setup, especially because two rounds of PCR are performed.

### Problem 7

Residual low-molecular-weight bands or primer carryover after enrichment and final purification (related to step-by-step method details, steps 23–24 and 35).

Residual primers or short DNA fragments may remain visible after enrichment PCR and may still be detected on agarose gels or TapeStation profiles.

### Potential solution

• Ensure that both 1× AMPure cleanups are performed carefully, and that ethanol is completely removed before elution.
• Confirm that the second size selection is carried out accurately to reduce the carryover of short fragments before PCR enrichment.
• Evaluate the enriched libraries on a 1.5% (w/v) agarose gel before and after the final cleanup to monitor primer removal.
• If low-molecular-weight peaks remain prominent and there is low enrichment within the 250–600 bp range, additional optimization of size selection or cleanup conditions may be required.

### Problem 8

Final library volume is insufficient for sequencing submission after quality-control assays (related to step-by-step method details, related to steps 23–25).

The final elution volume in this protocol is 30 μL. If the sequencing provider requires ≥25 μL of library pool, using library aliquots for multiple QC assays may leave insufficient volume for submission.

### Potential solution

• Confirm with the sequencing facility whether the remaining volume can still be accepted or whether concentration-based normalization by the facility is possible.
• Prioritize TapeStation or Bioanalyzer analysis when volume is limiting, because these systems require low input volume and provide both fragment-size distribution and an estimated library concentration.

## Resource availability

### Lead contact

Further information and request for resources should be directed to and will be fulfilled by lead contact, César Herrero-González (c.h.gonzalez@nhm.uio.no).

### Technical contact

Technical questions on executing this protocol should be directed to and will be answered by the technical contact, César Herrero-González (c.h.gonzalez@nhm.uio.no).

### Materials availability

This study generated custom barcoded adapters and primer combinations for MCSeEd library preparation. Sequences of these oligonucleotides are provided in the supplemental information. Additional information is available from the lead contact upon request.

### Data and code availability

Representative data generated in this study are included in the manuscript and supplemental information. Additional source data are available from the corresponding author upon request. The study did not generate code.

## Acknowledgments

This work was supported by the Highgrass project, led by Dr. Marian Schubert, which covered the acquisition of materials required for this protocol and the rental of the TapeStation system. We thank the Centre for Integrative Genetics (CIGENE) core facility at the 10.13039/501100008119Norwegian University of Life Sciences (10.13039/501100008119NMBU) in Ås, Norway, for providing access to the magnetic plate and for facilitating the use of the TapeStation instrument.

We also wish to thank Dr. Siri Birkeland (Natural History Museum, 10.13039/501100005366University of Oslo) for her interest in this protocol and for her helpful and constructive comments, which greatly improved the quality and readability of the manuscript.

## Author contributions

C.H.-G. carried out the library preparation workflow, including protocol implementation, optimization, troubleshooting, and manuscript preparation. S.S.J. contributed practical guidance during the initial stages of library preparation based on prior experience with this type of workflow, assisted with protocol planning, and troubleshooting. R.S.S. contributed to protocol optimization and troubleshooting by proposing freeze-and-squeeze gel recovery as an alternative to gel extraction kits, by suggesting a controlled two-step PCR enrichment strategy, and contributed to troubleshooting and manuscript preparation. M.S. supervised the project, oversaw library preparation, contributed to troubleshooting, and assisted in the design and preparation of the barcoded adapters used in this protocol.

## Declaration of interests

The authors declare no competing interests.
