# Supplementary material for: Protocol for optimized methylation context-sensitive enzyme ddRAD library preparation from partially degraded genomic plant DNA
Source: STAR Protoc. 2026 Aug 11;7(3):104778. doi: 10.1016/j.xpro.2026.104778 (PMC13487831; doi:10.1016/j.xpro.2026.104778)
Supplement: Supplementary file 1 [file mmc1.pdf]

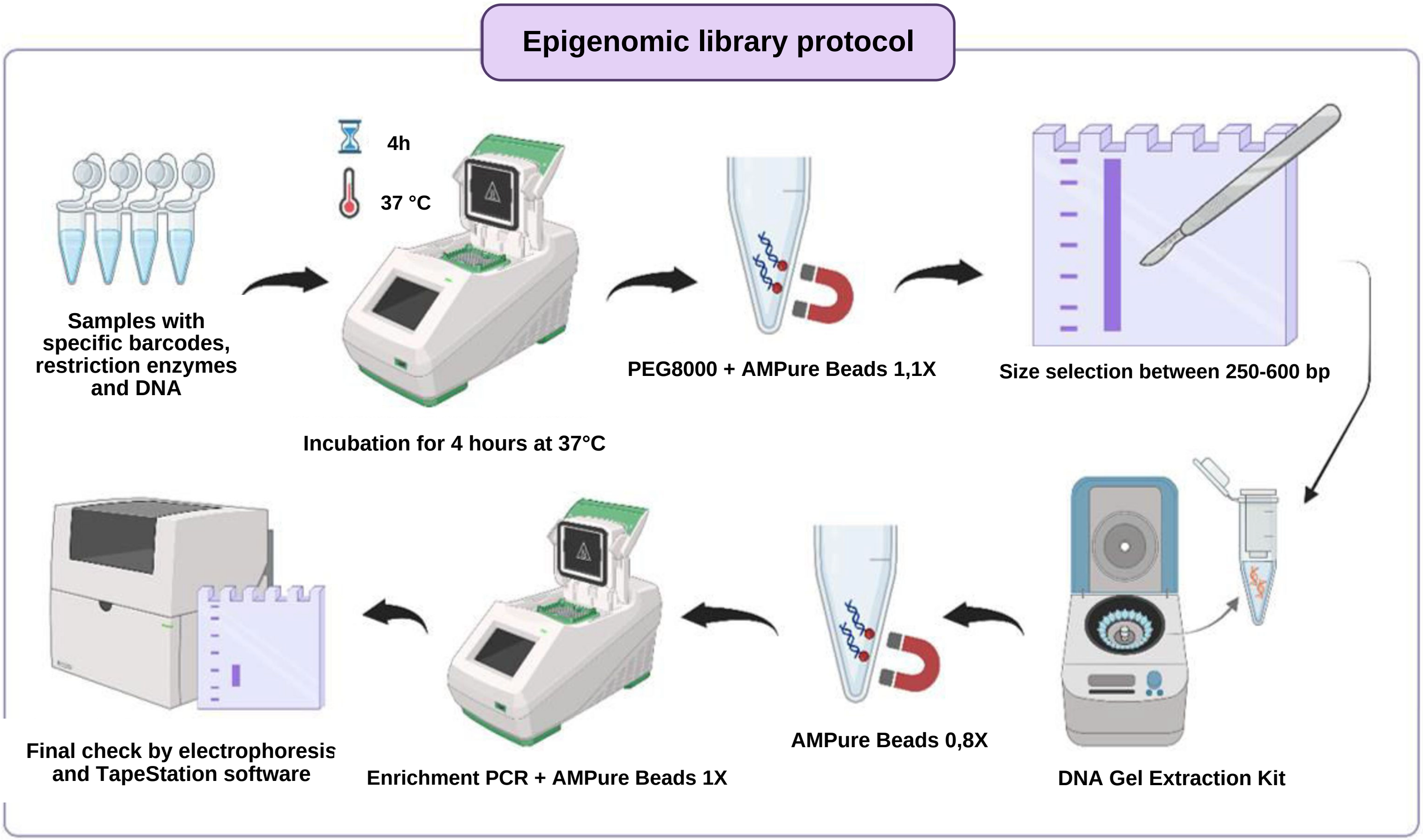
Epigenomic library protocol
4h
37 °C
Samples with specific barcodes, restriction enzymes and DNA
Incubation for 4 hours at 37°C
PEG8000 + AMPure Beads 1,1X
Size selection between 250-600 bp
DNA Gel Extraction Kit
AMPure Beads 0,8X
Enrichment PCR + AMPure Beads 1X
Final check by electrophoresis and TapeStation software
