# Supplementary material for: Protocol for optimized methylation context-sensitive enzyme ddRAD library preparation from partially degraded genomic plant DNA
Source: STAR Protoc. 2026 Aug 11;7(3):104778. doi: 10.1016/j.xpro.2026.104778 (PMC13487831; doi:10.1016/j.xpro.2026.104778)
Supplement: Supplementary file 2 [file mmc2.pdf]

Table S1, related to Before You Begin step 4. Barcodes developed for the preparation of epigenomic libraries, consisting of 10 samples per library for subsequent sequencing by MCSeEd. For each methylation-sensitive restriction enzyme, 20 adapters are included (10 forward and 10 reverse). These adapters share a common sequence but differ in their unique seven-nucleotide barcode, highlighted in bold. This table also includes the common MseI adapter, the common PCR primer (PCR1_MCSeEd) and 10 indexed PCR2 Illumina primers, each carrying a unique eight-base index sequence, also highlighted in bold.

Barcode adapter	Barcode ID	Adapter sequence
**P1**	PstI_P1_TOP	ACACTCTTTCCCTACACGACGCTCTTCCGATCT**GCTACAA**TGCA
	PstI_P1_BOT	[Phos] **TTGTAGC**AGATCGGAAGAGCGTCGTGTAGGGAAAGAGTGT
**P2**	PstI_P2_TOP	ACACTCTTTCCCTACACGACGCTCTTCCGATCT**AGCATCA**TGCA
	PstI_P2_BOT	[Phos] **TGATGCT**AGATCGGAAGAGCGTCGTGTAGGGAAAGAGTGT
**P3**	PstI_P3_TOP	ACACTCTTTCCCTACACGACGCTCTTCCGATCT**CCATCCA**TGCA
	PstI_P3_BOT	[Phos] **TGGATGG**AGATCGGAAGAGCGTCGTGTAGGGAAAGAGTGT
**P4**	PstI_P4_TOP	ACACTCTTTCCCTACACGACGCTCTTCCGATCT**ATCACGA**TGCA
	PstI_P4_BOT	[Phos] **TCGTGAT**AGATCGGAAGAGCGTCGTGTAGGGAAAGAGTGT
**P5**	PstI_P5_TOP	ACACTCTTTCCCTACACGACGCTCTTCCGATCT**CGATGTA**TGCA
	PstI_P5_BOT	[Phos] **TACATCG**AGATCGGAAGAGCGTCGTGTAGGGAAAGAGTGT
**P6**	PstI_P6_TOP	ACACTCTTTCCCTACACGACGCTCTTCCGATCT**TTAGGCA**TGCA
	PstI_P6_BOT	[Phos] **TGCCTAA**AGATCGGAAGAGCGTCGTGTAGGGAAAGAGTGT
**P7**	PstI_P7_TOP	ACACTCTTTCCCTACACGACGCTCTTCCGATCT**TGACCAA**TGCA
	PstI_P7_BOT	[Phos] **TTGGTCA**AGATCGGAAGAGCGTCGTGTAGGGAAAGAGTGT
**P8**	PstI_P8_TOP	ACACTCTTTCCCTACACGACGCTCTTCCGATCT**ACAGTGA**TGCA
	PstI_P8_BOT	[Phos] **TCACTGT**AGATCGGAAGAGCGTCGTGTAGGGAAAGAGTGT
**P9**	PstI_P9_TOP	ACACTCTTTCCCTACACGACGCTCTTCCGATCT**GCCAATA**TGCA
	PstI_P9_BOT	[Phos] **TATTGGC**AGATCGGAAGAGCGTCGTGTAGGGAAAGAGTGT
**P10**	PstI_P10_TOP	ACACTCTTTCCCTACACGACGCTCTTCCGATCT**CAGATCA**TGCA
	PstI_P10_BOT	[Phos] **TGATCTG**AGATCGGAAGAGCGTCGTGTAGGGAAAGAGTGT
**Barcoded adapters for enzyme *AciI***		
**P11**	AciI_P11_TOP	ACACTCTTTCCCTACACGACGCTCTTCCGATCT**CCACCGT**
	AciI_P11_BOT	[Phos] CG**ACGGTGG**AGATCGGAAGAGCGTCGTGTAGGGAAAGAGTGT
**P12**	AciI_P12_TOP	ACACTCTTTCCCTACACGACGCTCTTCCGATCT**CGTGCGA**

	AciI_P12_BOT	[Phos] CG**TCGCACG**AGATCGGAAGAGCGTCGTGTAGGGAAAGAGTGT
**P13**	AciI_P13_TOP	ACACTCTTTCCCTACACGACGCTCTTCCGATCT**GAACAAT**
	AciI_P13_BOT	[Phos] CG**ATTGTTC**AGATCGGAAGAGCGTCGTGTAGGGAAAGAGTGT
**P14**	AciI_P14_TOP	ACACTCTTTCCCTACACGACGCTCTTCCGATCT**ACTTGAA**
	AciI_P14_BOT	[Phos] CG**TTCAAGT**AGATCGGAAGAGCGTCGTGTAGGGAAAGAGTGT
**P15**	AciI_P15_TOP	ACACTCTTTCCCTACACGACGCTCTTCCGATCT**GATCAGA**
	AciI_P15_BOT	[Phos] CG**TCTGATC**AGATCGGAAGAGCGTCGTGTAGGGAAAGAGTGT
**P16**	AciI_P16_TOP	ACACTCTTTCCCTACACGACGCTCTTCCGATCT**TAGCTTA**
	AciI_P16_BOT	[Phos] CG**TAAGCTA**AGATCGGAAGAGCGTCGTGTAGGGAAAGAGTGT
**P17**	AciI_P17_TOP	ACACTCTTTCCCTACACGACGCTCTTCCGATCT**GGCTACA**
	AciI_P17_BOT	[Phos] CG**TGTAGCC**AGATCGGAAGAGCGTCGTGTAGGGAAAGAGTGT
**P18**	AciI_P18_TOP	ACACTCTTTCCCTACACGACGCTCTTCCGATCT**CTTGTAA**
	AciI_P18_BOT	[Phos] CG**TTACAAG**AGATCGGAAGAGCGTCGTGTAGGGAAAGAGTGT
**P19**	AciI_P19_TOP	ACACTCTTTCCCTACACGACGCTCTTCCGATCT**AGTCAAC**
	AciI_P19_BOT	[Phos] CG**GTTGACT**AGATCGGAAGAGCGTCGTGTAGGGAAAGAGTGT
**P20**	AciI_P20_TOP	ACACTCTTTCCCTACACGACGCTCTTCCGATCT**AGTTCCG**
	AciI_P20_BOT	[Phos] CG**CGGAACT**AGATCGGAAGAGCGTCGTGTAGGGAAAGAGTGT
**Barcoded adapters for enzyme *NsiI***		
**P21**	NsiI_P21_TOP	ACACTCTTTCCCTACACGACGCTCTTCCGATCT**ATGTCAG**TGCA
	NsiI_P21_BOT	[Phos] **CTGACAT**AGATCGGAAGAGCGTCGTGTAGGGAAAGAGTGT
**P22**	NsiI_P22_TOP	ACACTCTTTCCCTACACGACGCTCTTCCGATCT**CCGATAT**TGCA
	NsiI_P22_BOT	[Phos] **ATATCGG**AGATCGGAAGAGCGTCGTGTAGGGAAAGAGTGT
**P23**	NsiI_P23_TOP	ACACTCTTTCCCTACACGACGCTCTTCCGATCT**GACACGT**TGCA
	NsiI_P23_BOT	[Phos] **ACGTGTC**AGATCGGAAGAGCGTCGTGTAGGGAAAGAGTGT
**P24**	NsiI_P24_TOP	ACACTCTTTCCCTACACGACGCTCTTCCGATCT**TCGGTTC**TGCA
	NsiI_P24_BOT	[Phos] **GAACCGA**AGATCGGAAGAGCGTCGTGTAGGGAAAGAGTGT
**P25**	NsiI_P25_TOP	ACACTCTTTCCCTACACGACGCTCTTCCGATCT**CCGTCCC**TGCA
	NsiI_P25_BOT	[Phos] **GGGACGG**AGATCGGAAGAGCGTCGTGTAGGGAAAGAGTGT
**P26**	NsiI_P26_TOP	ACACTCTTTCCCTACACGACGCTCTTCCGATCT**GTCCGCA**TGCA

	NsiI_P26_BOT	[Phos] **TGCGGAC**AGATCGGAAGAGCGTCGTGTAGGGAAAGAGTGT
**P27**	NsiI_P27_TOP<br>NsiI_P27_BOT	ACACTCTTTCCCTACACGACGCTCTTCCGATCT**GTGAAAC**TGCA<br>[Phos] **GTTTCAC**AGATCGGAAGAGCGTCGTGTAGGGAAAGAGTGT
**P28**	NsiI_P28_TOP<br>NsiI_P28_BOT	ACACTCTTTCCCTACACGACGCTCTTCCGATCT**GTGGCCT**TGCA<br>[Phos] **AGGCCAC**AGATCGGAAGAGCGTCGTGTAGGGAAAGAGTGT
**P29**	NsiI_P29_TOP<br>NsiI_P29_BOT	ACACTCTTTCCCTACACGACGCTCTTCCGATCT**GTTTCGG**TGCA<br>[Phos] **CCGAAAC**AGATCGGAAGAGCGTCGTGTAGGGAAAGAGTGT
**P30**	NsiI_P30_TOP<br>NsiI_P30_BOT	ACACTCTTTCCCTACACGACGCTCTTCCGATCT**CGTACGT**TGCA<br>[Phos] **ACGTACG**AGATCGGAAGAGCGTCGTGTAGGGAAAGAGTGT
**Common adapter**		
MseI	MseI_TOP<br>MseI_BOT	GTGACTGGAGTTCAGACGTGTGCTCTTCCGATCTG<br>CAGATCGGAAGAGCACACGTCTGAACTCCAGTCAC
**Common PCR primer**		
**Index**	PCR1_MCSeEd	AATGATACGGCGACCACCGAGATCTACACTCTTTCCCTACACGAC
**Rest of PCR primers**		
**Illumina Index**	PCR2_Index_1	CAAGCAGAAGACGGCATACGAGAT**ATCACGAC**GTGACTGGAGTTCAGACGTGTGC
**Illumina Index**	PCR2_Index_2	CAAGCAGAAGACGGCATACGAGAT**ACAGTGGT**GTGACTGGAGTTCAGACGTGTGC
**Illumina Index**	PCR2_Index_3	CAAGCAGAAGACGGCATACGAGAT**CAGATCCA**GTGACTGGAGTTCAGACGTGTGC
**Illumina Index**	PCR2_Index_4	CAAGCAGAAGACGGCATACGAGAT**ACAAACGG**GTGACTGGAGTTCAGACGTGTGC
**Illumina Index**	PCR2_Index_5	CAAGCAGAAGACGGCATACGAGAT**ACCCAGCA**GTGACTGGAGTTCAGACGTGTGC
**Illumina Index**	PCR2_Index_6	CAAGCAGAAGACGGCATACGAGAT**AACCCCTC**GTGACTGGAGTTCAGACGTGTGC
**Illumina Index**	PCR2_Index_7	CAAGCAGAAGACGGCATACGAGAT**CCCAACCT**GTGACTGGAGTTCAGACGTGTGC
**Illumina Index**	PCR2_Index_8	CAAGCAGAAGACGGCATACGAGAT**CACCACAC**GTGACTGGAGTTCAGACGTGTGC
**Illumina Index**	PCR2_Index_9	CAAGCAGAAGACGGCATACGAGAT**GAAACCCA**GTGACTGGAGTTCAGACGTGTGC

**Illumina Index**	PCR2_Index_10	CAAGCAGAAGACGGCATACGAGAT**TGTGACCA**GTGACTGGAGTTCAGACGTGTGC
